# EEG, behavioural and physiological recordings following a painful procedure in human neonates

**DOI:** 10.1038/sdata.2018.248

**Published:** 2018-11-13

**Authors:** Laura Jones, Maria Pureza Laudiano-Dray, Kimberley Whitehead, Madeleine Verriotis, Judith Meek, Maria Fitzgerald, Lorenzo Fabrizi

**Affiliations:** 1 Department of Neuroscience, Physiology, and Pharmacology, University College London, London WC1E6BT, United Kingdom; 2 Elizabeth Garrett Anderson Obstetric Wing, University College London Hospitals, London WC1E6DB, United Kingdom

**Keywords:** Sensory processing, Paediatric research

## Abstract

We present a dataset of cortical, behavioural, and physiological responses following a single, clinically required noxious stimulus in a neonatal sample. Cortical activity was recorded from 112 neonates (29–47 weeks gestational age at study) using a 20-channel electroencephalogram (EEG), which was time-locked to a heel lance. This data is linked to pain-related behaviour (facial expression), physiology (heart rate, oxygenation) and a composite clinical score (Premature Infant Pain Profile, PIPP). The dataset includes responses to non-noxious sham and auditory controls. The infants’ relevant medical and pain history was collected up to the day of the study and recorded in an extensive database of variables including clinical condition at birth, diagnoses, medications, previous painful procedures, injuries, and selected maternal information. This dataset can be used to investigate the cortical, physiological, and behavioural pain-related processing in human infants and to evaluate the impact of medical conditions and experiences upon the infant response to noxious stimuli. Furthermore, it provides information on the formation of individual pain phenotypes.

## Background & Summary

Worldwide 5–18% of babies are born before term age^1^ (37 weeks) and many remain in hospital for some time following birth (mean neonatal intensive care unit (NICU) stay, 9.1 days^2^). While in the NICU or other postnatal wards, neonates can undergo up to 12-14 tissue breaking procedures a day^2,3^, many with little or no analgesic therapy^4^. In the absence of self-report, infant pain is commonly assessed using various behavioural and physiological measures^5,6^. However, significant advancement in the understanding of processing of pain in the infant nervous system has come from several imaging techniques such as electroencephalography (EEG)^7–9^, near infra-red spectroscopy (NIRS)^10–12^, and functional magnetic resonance imagining (fMRI)^13,14^. In particular, the discovery and analysis of nociceptive event related potentials (nERPs) have provided new insights into neonatal brain processing of acute noxious events^7,9,15–19^.

Many factors affect an individual’s sensitivity to a noxious stimulus, even at the beginning of life. Both the level of stress^8^ and the sex of the infant^20^ have recently been shown to be factors determining pain-related activity in the brain. During their hospital stay, the severity of illness influences neonatal pain-related behaviour^21^: a diagnosis of jaundice can result in delayed cortical somatosensory processing^22^, and infections can lead to an increase in pain sensitivity^23,24^. The number of painful procedures that neonates are exposed to is correlated with their future brain maturation, pain-related behaviour, stress reactivity and cognitive development^25–30^. At the time when these experiences are occurring, in the final trimester and early postnatal period, the neonatal brain undergoes significant changes in connectivity and organisation, including in areas and circuits implicated in pain processing^31,32^. The gradual development from non-specific bursting activity in premature infants following a painful stimulus, to a pain-specific event-related potential (nERP)^15^ is likely to contribute to a critical window of development, when activity dependent plasticity may determine pain sensitivity in future life.

**Figure 1:**
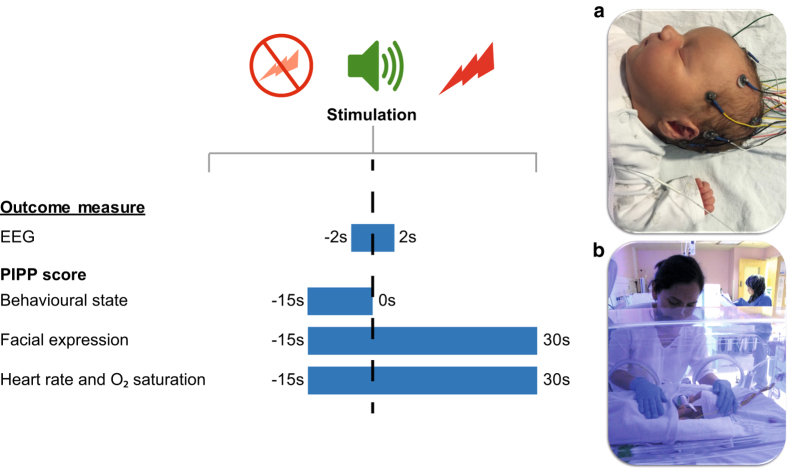
Study schematic. EEG and behavioural and physiological data for PIPP scoring were collected during sham control, auditory control, and noxious heel lance stimulations. (**a**) Infant at 41 weeks gestational age at study on the postnatal ward, with EEG electrodes placed according to a modified 10–10 system; (**b**) 34 weeks gestational age at study in special care.

The current dataset was collected as part of an ongoing study into the development of human neonatal pain processing. Cortical pain-related activity was recorded using EEG following a single noxious procedure (clinically required blood test)^8,15^. Facial expressions and physiological responses were also recorded as part of a standard composite pain score, the Premature Infant Pain Profile^33^ (PIPP; Fig. 1). Extensive notes were collected regarding the infant’s relevant medical and pain history up to the day of the procedure, as well as selected maternal information (Data Citation 1).

We present data from 112 infants (52 females; 29–47 weeks gestational age at study, 0.5–96 days postnatal age) recruited from the postnatal, special care, or intensive care wards at the Elizabeth Garrett Anderson Obstetric Wing, University College London Hospital (UCLH). The data includes 4 second epochs of a multi-channel EEG recording time-locked to heel lance, sham control, and auditory control stimuli, suitable for both ERP and time-frequency analysis. PIPP scores (plus the constituent facial expression and physiological scores) for each of the three stimuli are also provided. Moreover, information on over 100 variables has been collected from the patient notes. This information includes: the condition at birth, diagnoses, medications, previous painful procedures, and injuries. Data on maternal condition and medications is also provided.

This dataset is the first of its kind and is invaluable for those interested in pain processing in the human brain. The accompanying database of neonatal and maternal information combined with behavioural and physiological responses to both painful and control stimuli, allows initial exploration of factors that influence pain sensitivity.

## Methods

### Participants

**Figure 2:**
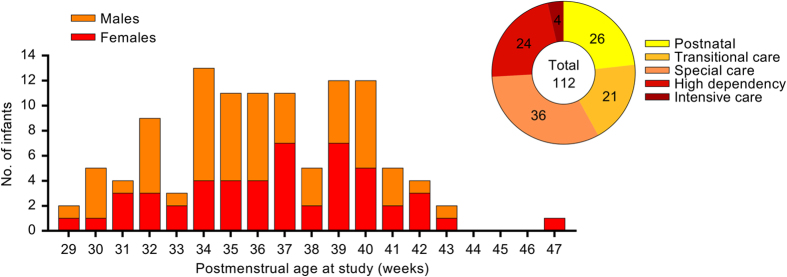
Gestational age at study, sex, and type of care of the infant patient sample. Gestational age at study in weeks represents a completed week, i.e. 29 = 29^+0^–29^+6^. Values in the pie chart are the number of babies in each type of care at the time of study.

One hundred and twelve neonates (Table 1) were recruited from the postnatal, special care, or intensive care wards at UCLH Elizabeth Garrett Anderson Wing (Fig. 2). The recruitment period was from June 2015 to January 2017. While this was an opportunity sample, infants were excluded if they had undergone therapeutic cooling or had Grade 4 Hypoxic Ischemic Encephalopathy (HIE), as they were too unstable for the EEG study. Written informed consent was obtained from the parents prior to each study. Additional informed consent was obtained from each parent for the use of identifiable images or videos in publications. The study was approved by the NHS Health Research Authority (London – Surrey Borders) and conformed to the standards set by the Declaration of Helsinki.

### Experimental design

Brain activity in response to noxious, sham control, and auditory control stimuli were monitored using EEG^8,9,15^. The noxious stimulus was a clinically required heel lance used for blood sampling performed by an experienced research nurse and time-locked to the ongoing EEG recording. To determine the PIPP score following the stimuli, the infant’s pulse, blood oxygen saturation and facial expression were monitored using a pulse oximeter and a video camera^16,33,34^. The research nurse recorded the infants’ relevant medical and pain history retrospectively from the patient notes, up to the day of the study.

### Stimulation

All stimuli (heel lance, sham control, auditory control) were performed using a lancet. The heel lance was always performed last to minimise any discomfort. The auditory control preceded the sham control stimulus in 24/99 trials. The order of stimulation for each study is provided. Each stimulus was time-locked to the ongoing EEG recording using an accelerometer mounted onto the lancet, which detects the vibrations of the blade being released^35^. Using a custom-made trigger box, the ongoing EEG is then marked at the time of blade release (Supplementary video 1).

1. *Heel lance*: The noxious stimulus was a heel lance that was clinically required to collect a blood sample and is considered as a mild painful stimulus by adults (average pain score of 2/10 from 367 adults^36^). Lances were never conducted for the sole purpose of the study, were performed by a trained nurse using a disposable lancet, and standard hospital practice was followed at all times. The heel was cleaned with sterile water using sterile gauze and the lancet placed against the heel for at least 30 s prior to the release of the blade. This was to obtain a baseline period free from other stimulation for both the EEG and PIPP. The heel was then squeezed 30 s after the release of the blade, again to ensure a post-stimulus period free from other stimuli. Babies were soothed as and when required. Parents were informed that they could hold their baby if they wished and babies were fed on demand throughout the study.
2. *Sham Control*: The sham control stimulus was obtained in the same conditions as the lance, but in this case the blade was facing away from the heel upon its release. Thus the vibration and acoustic stimulus caused by the blade release occurs, but with no skin-break.
3. *Auditory control*: The auditory control stimulus was obtained in the same conditions as the lance, but in this case the lancet was held near the foot without contact. Previous studies have used both the sham and auditory controls to ensure that the recorded brain activity during the noxious heel lance is specific to the skin-break^9,16^.

For each study we recorded the time of each stimulation, the stimulation site (i.e. left heel), the position and location of the infant, and the vigilance state based on behaviour, and the respiratory and EEG recordings (determined using 30 second epochs pre- and post-stimulation). During the study 53 (47%) babies were being held by a parent and 59 (53%) were in their cot. Of the babies held by a parent, 30 (57%) were prone against the parents chest, 22 (42%) were supine or lying on their side in the parents arms, and 1 (2%) was sitting upright. Prior to the heel lance, 3 (3%) infants were classified as active awake, 11 (10%) quiet awake, 55 (49%) active sleep, and 40 (38%) quiet sleep. Following the heel lance, 13 (12%) infants transitioned from sleep to wakefulness. The vigilance state of 1 infant could not be determined. The reason for the heel lance blood test (i.e. glucose), the quantity of blood required, and any distraction techniques used were recorded.

### Electroencephalography

#### EEG recording

EEG was recorded from up to 20 electrodes (disposable Ag/AgCl cup electrodes) in addition to the ground and reference electrodes. Recording electrodes were positioned according to a modified international 10/10 electrode placement system, with high density central-parietal and posterior temporal coverage, overlying primary visual (O1, O2), primary auditory (T7, T8), association (F7, F3, F4, FCz, F8, P7, P8, TP9, TP10, POz), and somatosensory cortices (C3, Cz, C4, CP3, CPz, CP4). The reference electrode was placed at Fz and the ground electrode at either FC1 or FC2 (depending on the position of the infant). Lead I electrocardiogram (ECG) was recorded from electrodes on both shoulders and respiratory movements were monitored with a movement transducer on the abdomen. Five ECG recordings used a bipolar reference rather than Fz (right ECG referenced to the left ECG), thus these files only contain 1 ECG trace rather than a separate trace for the left and right ECG. Electrode/skin contact impedances were kept to a minimum by gently rubbing the skin with a prepping gel (NuPrep, Weaver & Co.) and then applying the electrodes with a conductive paste (10/20, Weaver & Co.). A soft bonnet was then secured over the electrodes. EEG activity, from DC to ≥500 Hz, was recorded using the Neuroscan SynAmps2 EEG/EP recording system. Signals were digitised with a sampling rate of 2 kHz and a resolution of 24 bit. EEG was assessed by an experienced clinical scientist. All infants tested had an EEG within normal limits for their gestational age at study based on age-appropriate features, synchrony and symmetry of EEG activity between hemispheres, and absence of electrographic seizures^37,38^.

#### Segmentation

The EEG recordings were converted into EEGLAB (Swartz Center for Computational Neuroscience) data structures and segmented into 4 second epochs (2 seconds pre- and post-stimulus). No pre-processing was performed except for one file (253501L01) which was downsampled from 5 kHz to 2 kHz using EEGLAB. Each file contains the data from the EEG electrodes, ECG, and respiration transducer, with a trigger (denoted with “4” or “8”) indicating the onset of the stimulus at 0 s. Heel lance epochs are available for all 112 subjects, sham and auditory controls are available for 99 subjects. Missing control trials are due to: 1) control stimulation being added to the protocol after a pilot phase, 2) technical issues or the infant becoming restless during recording.

### Recording behavioural and physiological data

Infant facial expression was recorded on video and synchronised with the EEG recording with an LED light placed within the frame and activated by the blade release (Supplementary video 1). Beat-by-beat blood oxygenation and heart rate were monitored with a pulse oximeter (Nellcor Oximax) using a flexible infant probe wrapped around the lateral aspect of the non-stimulated foot and held in place using a soft Velcro strap. Using a custom MATLAB script data was stored and marked at the time of the heel lance trigger.

### Premature Infant Pain Profile

The Premature Infant Pain Profile (PIPP) is a commonly used clinical pain scoring system^33^. PIPP scores were calculated following the heel lance and the two control stimulations by combining facial expression and physiological measures. The facial expression score includes the percentage of time (out of 30 s) that a brow bulge, eye squeeze, and naso-labial furrow are exhibited during the 30 s post-stimulus period. A score of 1–3 is given for each facial expression and then combined. It is also noted if any of these facial expressions were present during the 15 s baseline period. For the physiological measure, the heart rate and blood oxygenation scores were determined by the difference between the mean values during the pre-stimulus period (15 s) and the maximum or minimum (respectively) values 30 s post-stimulus, again a score of 1-3 is given for each. The vigilance state at baseline was also assessed behaviourally, and classified as either active awake, quiet awake, active sleep or quiet sleep. The maximum PIPP score, obtained by summing the facial expression, physiological, and behavioural state scores, and adjusting for gestational age at study, is 21 with 0–6 indicating minimal/no pain, 7–12 slight/moderate pain, and >12 severe pain (for original description of the scoring procedure see^33^). The PIPP score is not available for all infants due to the infant moving out of the video frame, infant obscuring face with arms, or technical issues resulting in physiological data not being marked by a trigger. There are 79, 67, and 70 complete PIPP scores for the noxious heel lance, sham control, and auditory control stimuli, respectively.

### Patient notes

#### Infant notes

**Figure 3:**
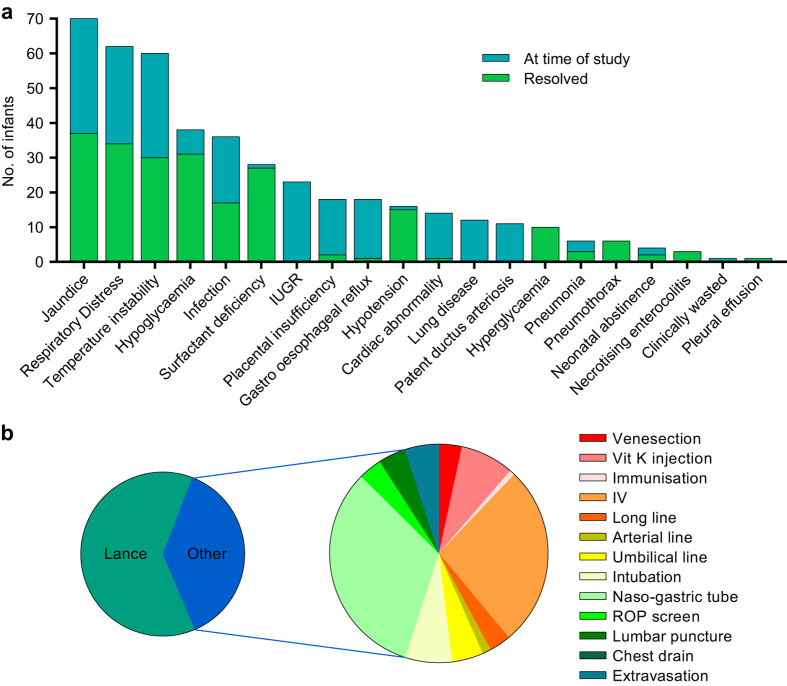
Diagnoses and procedures. (**a**) Frequency of each diagnoses within the sample. This includes those that were resolved (at least 3 days prior to day of study) and those that were ongoing around time of study (within 3 days prior to, or on the day of the study). Diagnoses with a frequency of 0 are not included (hypertension, adrenal insufficiency, hypothyroidism, and seizures). (**b**) Percentage of painful procedures. Lance (62%) and all other (38%) out of a total of 3661 procedures across all the subjects within the sample.

Detailed notes were retrieved from several sources: (1) Nursing charts logging blood gas results, serum bilirubin levels (SBR), haematology results, microbiology results, observations chart (e.g. vital signs and urine output), feeding times and volume, and medications chart. (2) Cranial ultrasound and other imaging results (e.g. MRI). (3) Echocardiography results. (4) Specialist reports in case of referrals, from within or outside the department. (5) Contemporaneous notes (logging of each event as and when it occurs). All sources were used to record ongoing or resolved diagnoses and painful procedures up to the day of the study (Fig. 3). Previous heel lances were also recorded. No infants in this sample had undergone surgery prior to the day of study.

#### Maternal notes

Following additional consent from parents we recorded the mother’s medications and any medical conditions. This information was collected using the electronic drug charts and maternal medical notes or provided by the midwives if these were not available. Maternal information was only collected if the baby was <3 days old or breast feeding and therefore likely to be directly influenced by such factors.

### Code availability

Custom written MATLAB code was used to export and store the data from the pulse oximeter. This code is specific to the data produced by the Nellcor N-560 pulse oximeter using MATLAB version R2011b (The Mathworks, MA, USA), and is available upon request from the corresponding author. This code exports the pulse rate and blood oxygen saturation percentage every 2 s into a .txt file.

## Data Records

### Data retrieval

All the data described can be downloaded from ReShare (Data Citation 1). This includes: 4 s raw EEG files for the heel lance, sham control, and auditory control stimuli, infant demographics, details of the EEG recording, stimulation information including PIPP scores, infant patient notes including pain history and relevant medical information, and maternal patient notes. This data contains sensitive medical information, although anonymised. For this reason, access to the data will be under the terms and conditions for safeguarded data (as detailed in ReShare: http://reshare.ukdataservice.ac.uk/legal/#Safeguarded) but is available upon request to any medical or research professional.

Also available with these data records is a summary of the methods and a detailed description of the organisation and contents of the EEG files and pain and medical history database.

### Data format and organisation

A parent folder (“Database”) contains excel spreadsheets pertaining to “Infant demographics”, “Study details”, “Stimulation information”, “Infant patient notes”, and “Maternal patient notes”. Table 2 provides the names of the sheets within each excel file. The first column in each sheet contains the “record identifier”, which is unique for each infant studied and links the information across all the sheets and excel files. The “Stimulation information” file has a sheet for each of the 3 stimulation types and the second column of each of these sheets contains the name of the infant’s EEG file (“EEG file name”). This file name is the record identifier, appended with the stimulation type identifier: L01 (heel lance), C01 (sham control), or A01 (auditory control). One sham control file is named “C02” as the first event failed to trigger and was therefore repeated. The time difference between the first and second attempt was 2 minutes. The EEG files are stored in folders by infant (112 folders) within a second parent folder “EEG” (<EEG file name>.mat).

## Technical Validation

### Electroencephalography

EEG was recorded according to clinical standards by an experienced clinical scientist. Clinical guidelines suggest recording ECG and respiratory data with the EEG. The ECG highlights episodes of tachycardia and bradycardia which are common in pre-term infants, with bradycardia episodes often associated with apnoea and desaturation^39,40^. Both ECG and respiratory data improve the detection of artefacts such as associated with heart rate or widespread movement^41^, and inform infant sleep staging^42,43^.

We adopted a protocol optimised for the acquisition of EEG within a hospital ward environment. The research nurse wore anti-static nursing clogs (ToffeIn) and apron. The clinical scientist monitored the EEG throughout the study and only signalled the nurse to proceed when the EEG was clear of artefacts. EEG characteristics, such as the presence of delta brushes, were assessed and have been made available in the “Study details”.

**Figure 4:**
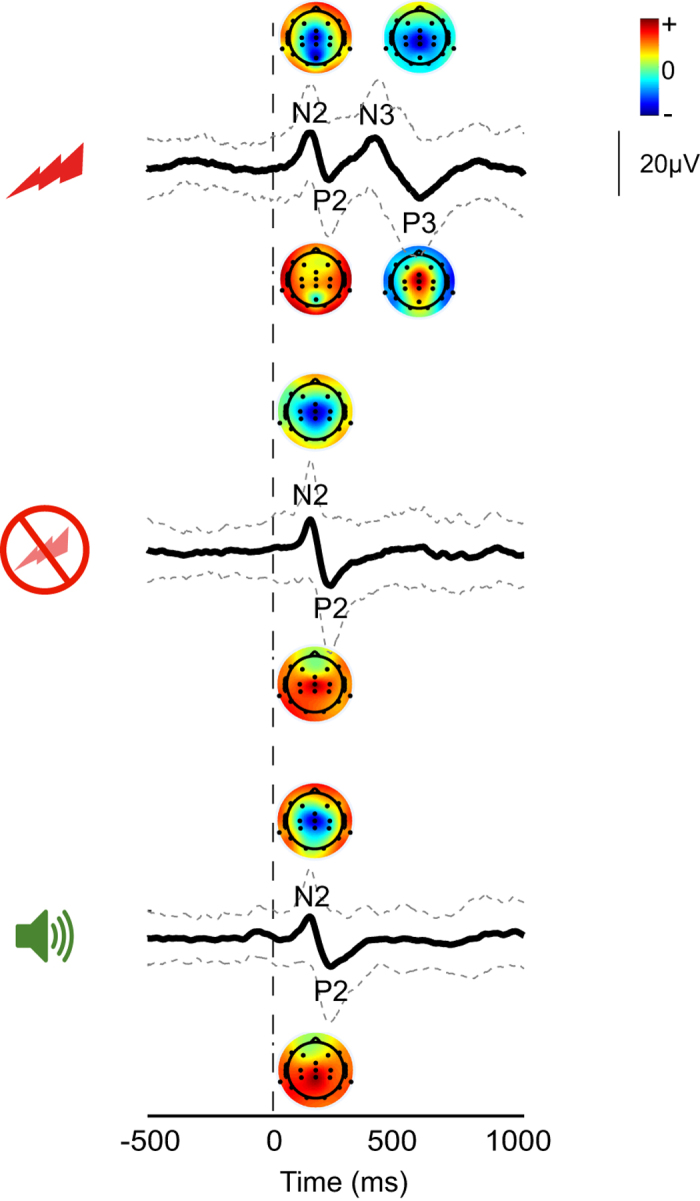
Average waveforms and topographical plots. Group average waveforms at Cz and corresponding topographic maps for each ERP following heel lance (top), sham control (middle), and auditory control (bottom) stimulation. Stimulus onset at 0 ms is marked by the vertical black dashed line. Standard deviation is represented by the grey dashed lines.

To assess the quality of the data and potential pain-related ERP, using MATLAB and EEGLAB, raw data were filtered with second-order bidirectional Butterworth bandpass (1–30 Hz) and notch (48–52 Hz) filters and epoched between 0.5 s prior to and 1 s following the stimulus. Baseline correction was carried out using the pre-stimulus interval. Epochs contaminated with movement artifact (signal exceeding ± 100 μV) or delta brush activity (characterised by high voltage delta activity with over-riding alpha-beta oscillations, typically ranging between 50 and 300 μV^44^), around the time of the nERP were removed. Figure 4 shows the average ERP response at Cz following the heel lance, sham control, and auditory control stimulation. The N2 and P2 component of the ERP can be seen in all three average waveforms, but the pain-related ERPs (N3 and P3) only following the noxious heel lance^9^.

### Premature infant pain profile (PIPP)

The physiological score of the Premature Infant Pain Profile (PIPP) does not require technical validation because it is directly calculated from data collected using clinical standards (pulse oximetry). Facial expression scoring was performed off site using the video recording of the infant’s face. This allowed for repeated viewing and more accurate scoring. Videos were scored by a single neonatal research nurse for consistency. The nurse was trained by a post-doctoral researcher with experience in PIPP scoring using a 5 trials sample.

According to the PIPP, 60% of infants had a mild to no pain response to heel lance, 34% moderate, and 6% severe. The average PIPP score following the heel lance is significantly higher than control scores (mean = 7, range = 2–17; sham: *t* = 6.40, *p* < 0.001; auditory: *t* = 5.94, *p* < 0.001), which are not significantly different from each other (sham: mean = 4, range = 1–10; auditory: mean = 4, range = 1–13; *t* = 0.61, *p* = 0.546). The range of PIPP scores following the heel lance is consistent with previous reports^34,45^.

### Simultaneous recordings

The system that simultaneously marks EEG, pulse oximetry and video has been extensively tested for accuracy^35^ and used in previous research^8,46^. The system can detect the release of the blade from the lancet with 100% sensitivity and specificity and the timing of the stimulus is determined with a precision of 33 μs.

### Patient notes

Pain history and relevant medical information were obtained from the patient notes and collected by an experienced nurse. Whenever possible, missing information or diagnoses requiring follow up tests were collected from the notes at a later date.

## Additional information

**How to cite this article**: Jones, L. *et al*. EEG, behavioural and physiological recordings following a painful procedure in human neonates. *Sci. Data*. 5:180248 doi: 10.1038/sdata.2018.248 (2018).

**Publisher’s note**: Springer Nature remains neutral with regard to jurisdictional claims in published maps and institutional affiliations.

## Supplementary Material



Supplementary Video 1

## Figures and Tables

**Table 1:** Infant demographics.

Gestational age at birth (weeks ^+ days^)	35^+2^ (23^+2^–42^+4^)
Gestational age at study (weeks ^+ days^)	36^+4^ (29^+3^–47^+6^)
Postnatal age (days)	5 (0–96)
No. female	52 (46%)
No. multiple births (twins)	17
Birth weight (g)	2270 (480–4592)
No. caesarean deliveries	55
Apgar score^∗^ (5 min)	9 (4–10)
Duration of mechanical ventilation (days)	0 (0–36.7)
Values represent the median and range. ^∗^A simple and quick assessment, scored out of 10, to determine if a newborn requires any medical intervention immediately at birth. Gestational age at birth is the number of weeks from the first day of the mothers last menstrual cycle to the birth, and gestational age at study (also referred to as postmenstrual age) is the gestational age at birth plus the number of days since birth (postnatal age)^47^.	

**Table 2:** Names of database files and sheets.

Excel file name	Sheets	Examples of Information included
Infant demographics	Demographics	Gestational age, sex, weight, size.
	Delivery details	Resuscitation, multiple births, Apgar score.
	SNAP scores	Measures and final scores^48^.
Study details	Study context	Study duration, ward, feeding.
	EEG details	Recorded electrodes, occurrence of delta brushes, if normal for gestational age at study.
Stimulation information	Heel lance	Stimulation site, infant position, vigilance state, PIPP score.
	Sham control	
	Auditory control	
Infant patient notes	Ventilation	Type and number of days of ventilation.
	Diagnosis	Current and resolved diagnoses, 28 diagnoses recorded. See Fig. 3.
	Scans	Results of cranial ultrasound and MRI.
	Medication	Medications taken up to time of study, time since last dose.
	Heel lances	Visible marks on heel, total no., bruising on heel.
	Painful procedures	Occurrence of 16 different painful procedures, and time since most recent.
	Injuries	Fractures, burns, detailed notes of treatment.
Maternal patient notes	Maternal	Medications and conditions during pregnancy.
Due to the large numbers of variables collected, in rare instances the information was not available for an infant and appear as blank cells in the database. Infants admitted to the neonatal unit that are severely ill at birth have additional physiological factors recorded in order to calculate a SNAP score (for example: serum pH, blood pressure, and temperature). This is used to assess infant illness severity and risk. A SNAP II and SNAPPE II score is available for 28 and 27 babies, respectively.		
