# Author Correction: EEG, behavioural and physiological recordings following a painful procedure in human neonates

**DOI:** 10.1038/s41597-023-02346-1

**Published:** 2023-07-27

**Authors:** Laura Jones, Maria Pureza Laudiano-Dray, Kimberley Whitehead, Madeleine Verriotis, Judith Meek, Maria Fitzgerald, Lorenzo Fabrizi

**Affiliations:** 1 Department of Neuroscience, Physiology, and Pharmacology, University College London, London, WC1E6BT UK; 2 Elizabeth Garrett Anderson Obstetric Wing, University College London Hospitals, London, WC1E6DB UK; 3 Present Address: Developmental Neurosciences Program, University College London Great Ormond Street Institute of Child Health, London, WC1N1EH UK

**Keywords:** Databases, Anatomy, Biomarkers, Cardiovascular biology, Circulation

Correction to: *Scientific Data* 10.1038/sdata.2018.248, published online 13 November 2018

The original corresponding author of the work, Dr Laura Jones, has left UCL is no longer able to respond to correspondence relating to this paper. Please contact Dr Lorenzo Fabrizi (l.fabrizi@ucl.ac.uk) for any queries, including requests for data access, rather than the listed corresponding author in the paper.

